# Supplementary material for: Mental health problems and admissions to hospital for accidents and injuries in the UK military: A data linkage study
Source: PLoS One. 2023 Jan 24;18(1):e0280938. doi: 10.1371/journal.pone.0280938 (PMC9873160; doi:10.1371/journal.pone.0280938)
Supplement: Attachment — Submitted filename: Response to Reviewers.docx [file pone.0280938.s001.docx]

08 September 2022

Dear Dr Shepherd,

**Re:** PONE-D-21-32763

**Title:** Mental health problems and admissions to hospital for accidents and injuries in the UK military: A data linkage study

Thank you for sending us the reviewers’ comments and for considering this paper for publication in PLOS ONE. We have addressed the reviewers’ comments below and attach a copy of the revised manuscript.

**Major Revisions:**

1. **The title does not seem descriptive enough to me. The current title reads more like a methods paper to me. I do not see the data linkage and use of EHRs as the key aspect of the findings. This paper seems to be reporting how mental health and military characteristics are associated with a variety of injury and accident outcomes; I recommend working some version of that into the title.**

Thank you for this suggestion. The title has now been amended to reflect the key findings on mental health and hospital admissions for accidents and injuries (see line 1-2).

“Mental health problems and admissions to hospital for accidents and injuries in the UK military: A data linkage study”.

2. **Lines 97-99 seem too strong regarding deployment. There are many possibilities for why military personnel may have more injuries and higher risk taking than the general population. (Many of these are mentioned later in the Discussion). The military may enlist people with higher risk-taking due to their individual interests or the military’s recruitment strategies. Other military experiences and training may increase risk taking. Deployment is not the first thing that comes to mind. I recommend acknowledging several possibilities and then going on to discuss deployment as one possibility.**

Thank you for pointing this out. The statement has been amended to include other possibilities for why military personnel may have more injuries and higher risk taking than the general population (see line 78-80). This section now reads as:

“Accidental death or injury is more common in individuals who report higher levels of risk-taking behaviour, which may be more likely in military personnel than the general population as an indirect result of individual interests, military training or deployment experiences.”

We have also amended a statement in the abstract to clarify that risky behaviour in military personnel is not primarily due to deployment experiences (see line 32-35). This section now reads as:

“It is a common misconception in the general public that accidental injuries are always the result of random events, however research suggests that mental health problems and the increased levels of risky behaviour in military personnel may play a role.”

3. **I recommend re-naming “any mental health problem” to something like “Either CMD or PTSD,” since the authors did not assess for any mental health problem.**

Thank you for this suggestion. The variable “any mental health problem” has now been renamed to “CMD or probable PTSD” in the tables. We believe this modification has improved the readability of the manuscript.

4. **I was fairly late into the paper before I realized that self-inflicted injuries (i.e. suicidal behaviors) were included. Given the set-up of the paper, this surprised me, and I do not typically think of self-inflicted injuries in the same category as some of the accidents and other injuries. Is it possible that different results would be found with more specific categories?**

Thank you for highlighting this. We agree that self-inflicted injuries do not intuitively belong in the same category as some of the accidents and other injuries, and have addressed this in the limitations section (see line 421-424). This section now reads as:

“For this paper, hospital admissions were identified in healthcare records using the internationally recognised ICD-10 codes in Chapter XX “External causes of morbidity and mortality”. We acknowledge that this ICD-10 Chapter includes a range of differing outcomes.”

5. **There are so few accidents and injuries for most categories… I do not think there is anything the authors can do about this, but this reduces the impact of the paper.**

The authors thank the reviewer for pointing this out and agree that the number of admissions for accidents and injuries is low for most categories and some of the associations should be treated with caution due to small cell sizes. This is one of the limitations of using administrative data, which we have highlighted in the limitations section (see line 413-416). This section now reads as:

“Furthermore, some of the associations should be treated with caution due to small cell sizes, which is a limitation of using administrative data from electronic health records. As the first data linkage of this kind, we have published all outcomes relevant to the aims of this study whilst ensuring that reporting guidelines for administrative data were adhered to and individuals were not identifiable from small cell sizes.”

6. **Table 2 does not appear to indicate statistical significance as reported in the results (and the other tables).**

Thank you for bringing this to our attention. As Table 2 reports unweighted frequencies and weighted percentages rather than odds ratios, we have not indicated statistical significance. Furthermore, the majority of variables in this table have not been treated as exposure variables and the associations for the main exposure variables have been reported in Tables 3, 4 & 5. We acknowledge that the results in this table may be open to misinterpretation known as the “Table 2 Fallacy” whereby associations for covariates are mistakenly interpreted in the same way as associations for main exposure variables (Westreich & Greenland; 2013). To meet the requirements of the journal, we were not able to include another table for the regression analyses conducted for Table 2 and have instead reported the significant associations in the results section of the manuscript.

7. **Binge drinking results are reported for Table 2 in the Results section but I did not see it in Table 2.**

Thank you for bringing this to our attention. Due to the requirements of the journal, we were not able to include another table for the regression analyses conducted for Table 2 and have instead reported the significant associations in the results section of the manuscript. The associations for binge drinking with the main outcome variables (admission to hospital and number of A&E attendances) have been reported in Tables 3, 4 & 5.

8. **Table 2: For the 4th and 5th most common accidents and injuries (columns 4 and 5), the authors suppressed almost half the variables (5/12) due to low n’s. I understand this is important, but it raises the question of whether there is value to including all 5 accident and injury categories.**

Thank you for highlighting this. The authors acknowledge that the cell sizes are low for the 4^th^ and 5^th^ most common accidents and injuries. Whilst the suppression of numbers was unavoidable due to reporting guidelines for administrative data, we believe including all 5 categories is important given the significant associations for “Sequelae of external causes of morbidity and mortality” and “Intentional self-harm” in the unadjusted analyses, as shown in Table 4.

9. **Line 288, while it becomes clear that these findings are unadjusted, I recommend labeling them as unadjusted to avoid any confusion.**

Thank you for this suggestion. The statement has been amended to clarify that these findings are unadjusted (see line 294-297). This section now reads as:

“In the unadjusted model, those with mental health problems were 1.5 times more likely than those without mental health problems to have an admission to hospital for accidents and injuries (Table 3). This effect remained significant in all adjusted models. Alcohol misuse, binge drinking and smoking were not significantly associated with having an admission to hospital for accidents and injuries.”

10. **It would help if the authors provided power analyses to assist the reader in understanding the risk of type II error. I see in HRs >2.0 that were not significant. This appears to be a major limitation of the study.**

Thank you for bringing this to our attention. The authors acknowledge the limitations of using administrative data and reporting low numbers, as outlined in the limitations section. However, post-hoc power calculations are generally not recommended and seen as fundamentally flawed, with CIs and p values providing a more meaningful quantification of uncertainty (Lydersen, 2014).

11. **The authors did a good job describing other limitations in the manuscript. Some of these pose some risk to the results.**

The authors thank the reviewer for their comment and agree that the study is not without limitations, however we believe the findings add value to the current literature on the risk of accidents and injuries among UK military personnel. We acknowledge that a more comprehensive understanding of the relationships between mental disorders and accidental death is needed but we believe that the current study provides useful findings for future studies to build on.

**Minor Revisions:**

A. **The Abstract seems to set up post-deployment risky behavior as a key topic, but I do not think that is the focus of the manuscript. I recommend revision of the Purpose section.**

The authors thanks the reviewer for this suggestion and acknowledge that post-deployment risky behaviour is not a key focus of the manuscript. However, as a key finding of the paper showed that smoking was associated with an increased risk of being admitted to hospital for an accident or injury, we believe it is important to highlight the important role of post-deployment risk behaviours in the abstract.

B. **In the Introduction, can you clarify if the stats comparing the Armed Forces to the UK population were controlled for demographic differences?**

We can confirm that the statistics provided by the Ministry of Defence comparing the Armed Forces to the UK general population were age and gender standardised to the 2018 UK Regular Armed Forces population.

C. **I recommend adding the websites for government studies (e.,g references 1 & 2)**

Thank you for this suggestion. We have added the websites for government studies ([www.assets.publishing.service.gov.uk](http://www.assets.publishing.service.gov.uk)) in the references section for references 1 & 2.

D. **In the Demographics section (Line 151), I recommend describing the educational categories for the broad readership of the journal.**

Thank you for this suggestion. We have added further information about the education categories (see line 130-136). This section now reads as:

“Demographics: Age (at time of phase 2 questionnaire), relationship status and educational attainment (categorised as O-Levels/GCSE or below and A-Levels or higher) were assessed at phase 2. GCSE stands for “General Certificate of Secondary Education” and is an academic qualification in a particular subject, taken in England, Wales and Northern Ireland. Students study for their GCSE’s during Year 9 to Year 11, between the ages of 14 and 16. A-Levels (Advanced Levels) are a college or sixth form leaving qualification offered in England, Wales, and Northern Ireland. These are not compulsory, unlike GCSEs, but are generally required across the board for university entrance. A-Levels are taken in Year 12 and 13, between the ages of 16 to 19.”

E. **Center is misspelled for the National Center for PTSD (Line 162)**

Thank you for bringing this to our attention. This spelling mistake has now been amended (see line 150).

F. **The AUDIT acronym is not defined**

Thank you for highlighting this. The AUDIT acronym has now been defined (see line 156).

G. **Line 286 – compared to what group?**

Thank you for bringing this to our attention. The statement has been amended to clarify that the comparison groups were serving personnel, non-smokers and those without a mental health problem, respectively (see line 281-283). This section now reads as:

“Admissions for intentional self-harm were more common among ex-serving personnel, smokers and those with mental health problems compared to serving personnel, non-smokers and those without a mental health problem, respectively.”

H. **Line 348 - I do not think military leaders would agree that service members are encouraged to take risks during deployment. Safety is a major focus. I recommend clarifying or deleting.**

The authors thank the reviewer for this comment and agree that this statement should be deleted (line 396-398). This section now reads as:

“While taking risks may be more likely during deployment, it is important that military personnel returning to civilian life do not continue to engage in risk-taking behaviours that could result in accidental injury or taking longer to recover from injuries.”

We thank the editors and reviewers for their comments and advice regarding this paper. We hope that you find that these revisions address the reviewers’ comments suitably.
