# Supplementary material for: Mental health problems and admissions to hospital for accidents and injuries in the UK military: A data linkage study
Source: PLoS One. 2023 Jan 24;18(1):e0280938. doi: 10.1371/journal.pone.0280938 (PMC9873160; doi:10.1371/journal.pone.0280938)
Supplement: Attachment — Submitted filename: Response to Reviewers.docx [file pone.0280938.s002.docx]

28 December 2022

Dear Dr Shepherd,

**Re:** PONE-D-21-32763

**Title:** Mental health problems and admissions to hospital for accidents and injuries in the UK military: A data linkage study

Thank you for sending us the reviewers’ comments and for considering this paper for publication in PLOS ONE. We have addressed the reviewers’ comments below and attach a copy of the revised manuscript.

**Major Revisions:**

1. **I appreciate the authors adding the limitation in lines 413 about the small cell size. However, their statement that this is a limitation of administrative data from health records is not correct in my opinion. This is a limitation of the rarity of some outcomes and the size of the cohort available for study.**

The authors thank the reviewer for their comment. The statement has now been amended to clarify the reason for small cell sizes in the analyses (see line 421-422). This section now reads as:

“Furthermore, some of the associations should be treated with caution due to small cell sizes, which is a limitation of the rarity of some outcomes and the size of the cohort available for the study.”

2. **I appreciated the authors adding a limitation in Lines 421-424 about including a range of different outcomes. That did not address my original concern, however. I am wondering if there is a different pattern of results for self-inflicted injuries? Given the goals of the paper, should self-inflicted injuries be included with the other accidents and such in this way?**

Thank you for this suggestion. The authors acknowledge that intentional self-harm is very different to some of the other outcomes. However, the question of why intentional self-harm is included in the ICD-10 Chapter XX "External causes of morbidity and mortality" is beyond the scope of this paper, especially during the second round of reviews. A statement about this limitation has been added based on comments in the first round of reviews and to address this limitation further would entail restructuring the entire paper, which we don't believe is feasible at this stage.

3. **I appreciate that the authors do not want to report post-hoc power analyses, but their response did nothing to address a major limitation of the manuscript. Some analyses are not able to detect associations unless they are massive. Some of the CIs are massive. This limitation is not sufficiently addressed.**

Thank you for bringing this to our attention. A statement has now been added to address the risk of type II error in the analyses (see line 423-424). This section now reads as:

“As a result, some analyses produced only small effect sizes and small numbers may have increased the risk of type II error, also known as a false negative.”

4. **Nearly all the key findings are reporting SMALL effect sizes. This should be emphasized in the abstract, in the initial key findings section of the Discussion, and the conclusion.**

Thank you for this suggestion. The following statements in the abstract and discussion have now been amended to emphasize that the findings of this study report small effect sizes (see line 56-58; line 369-371). These sections now read as:

“The findings suggest that accidents and injuries among military personnel are not always random events and that there are health and behavioural factors, including poor mental health and smoking, which are associated (with small effect sizes) with an increased risk of being involved in an accident.” Line 56-58.

“The main findings of the current study show that, among UK military personnel, poor mental health and smoking are associated (with small effect sizes) with an increased risk of being admitted to hospital for an accident or injury.” Line 369-371.

5. **A thorough read to reduce language that hints at causation is needed. For example, lines 370-371; Line 376 states military personnel are not protected from accidents – the study did not asses this; Line 384-385; Line 386-387 say smoking may play an important role – that is not my read of the results and effect sizes; Lines 389-381 is a bit blunt and overstated; etc. I recommend a critical read by some of the authors who are sensitive to language appropriate for use in association studies.**

Thank you for highlighting this. The study timeline was designed to ensure that all exposures under study came before the outcomes. We have also accounted for potential confounders in these associations and avoided the use of the term "causality". However, the authors acknowledge this comment and the statements mentioned above have now been amended to reduce language that hints at causation.

“However, the current study has identified factors which are associated with an increased likelihood of accidents and injuries in military personnel, such as mental health problems.” Line 382-383.

“However, it is possible that these traits may lead to unnecessary or non-relevant forms of risk-taking, such as alcohol use or dangerous driving, once personnel return from deployment. These findings potentially have implications for the prevention of premature death among people with mental illness.” Line 389-392.

“Another risk factor for premature death is smoking (36) and our findings suggest that smoking is associated with accidents and injuries among military personnel (with small effect sizes).” Line 393-395.

6. **The word “may” is used throughout the Discussion to temper interpretations. That is needed but given some of the points that are made and the limitations of the study, I think more clear language is needed. For example, in some places, the authors should be explicit that they are speculating based on evidence from prior research. Such language would provide a more appropriate context for the discussion**

Thank you for this suggestion. The following statement has been amended to clarify that we are speculating based on evidence from prior research and not trying to hint at causation (see line 397-399).

“Based on evidence from previous research, we hypothesise that this combination of having poorer health and also engaging in health damaging behaviours may make smokers more likely to be involved in accidents.”

7. **Additional limitations should be noted:**
i. **The outcomes were only tracked through 2014. The data are fairly old and changes since then are possible.**
ii. **The variables used are SO BROAD. Hard to know what some results mean.**
iii. **The study methods are quite complex. The epidemiological implications and limitations of so many methodological decisions are unknown. The author team has experts in these areas – more epidemiological limitations should be highlighted in my opinion**

The authors thank the reviewer for their comments.

i. Unfortunately, the data is fairly old due to delays in the peer review process that were out of our control.
ii. The variables used have been taken from the KCMHR cohort study or the ICD-10 and the authors only strive to provide one interpretation of the results.
iii. We acknowledge that there are several epidemiological limitations with this study but it is beyond the scope of the paper to discuss these further or go into detail about methodological decisions.
8. **In the Methods where the linkage to health care records is described, it would be helpful to provide some assurances about how comprehensive these records are for the countries named. I confess this may only be a problem for non-UK readers, but in some countries, any attempt to link to health care records would include massive gaps and holes for a population study. If this includes ALL medical care in those countries, that would be a strength to describe in more detail.**

The authors thank the reviewer for pointing this out and a statement has now been added to emphasize that secondary healthcare is comprehensively recorded for England, Scotland and Wales. (see line 170-172). This section now reads as:

“Unlike many countries, secondary healthcare in England, Scotland and Wales is comprehensively recorded and publicly available through application.”

9. **The Discussion on intentional self-harm (lines 399-406) currently describes the unadjusted analyses as if they are the primary results. This is probably because they are in the expected direction. However, there is only a brief mention of the surprising results that the adjusted analyses were not significant… I recommend reversing the tone of the paragraph to emphasize the adjusted analyses and offering some possible explanations if they exist (e.g., power, something in the methods, something unique about the study topic that could lead to these unexpected results?)**

Thank you for pointing this out. The statement has now been amended to emphasize that the association did not remain significant in the adjusted analyses (see line 409-414). This section now reads as:

“The findings showed that having a mental health problem and smoking were only associated with intentional self-harm in unadjusted models but effects diminished after accounting for demographic and military characteristics so may have been be explained by other factors including age, relationship status, military characteristics or family relationship adversity (21, 48-50)”.

10. **Why weren’t participants who only took part at phase 1 included? Was it a consent issue? I recommend specifying (Line 128).**

Thank you for this suggestion. The statement has now been amended to clarify why participants who only took part in phase 1 were not included in the analyses (see line 127-132). This section now reads as:

“In total, 9,990 individuals completed the phase 2 questionnaire (overall response rate 56%) (6) and 86% of the phase 2 participants provided consent for linkage of their cohort data to healthcare records (n=8,602) (22). Individuals who took part in phase 1 only are not included in the analyses as they had not been asked for consent to access their medical records. The authors obtained section 251 approval of the National Health Service Act 2006 to ensure full compliance with standard ethical procedures.”

11. **I acknowledge this is probably too large of an issue to mention as a new topic in a second review, but in case it is helpful to the authors, I will mention that many readers (maybe most) will wonder whether there were different results for PTSD vs. CMD. They are VERY different. If there is a way to address this, even descriptively, I think readers would appreciate it.**

Thank you for this suggestion. The authors recognise that there are differences between CMD and PTSD, however PTSD can be categorised as a CMD according to some criteria (e.g. NICE Clinical Guideline CG123). To acknowledge the differences between these conditions, we have added the following statement to the limitations (see line 424-426).

“In terms of examining associations with mental health problems, the authors recognise that CMD and PTSD are different conditions but had to be combined due to small cell sizes.”

**Minor Revisions:**

1. **A) This study use multi scales and difference score range. Thus, the authors should show the standard score and criteria in any scale of this study. B) The data-based that is used in this study should show how select or screen before used in this study. Moreover, the authors should explain how to validate the data for this study. C) Some scales are binary and some scales are interval. The authors should check the data distribution before using logistic analysis.**

Thank you for highlighting this. The authors have outlined in the measures section where outcome data were categorical or continuous. The distribution of continuous data was checked and appropriate regression approaches (e.g. binomial) were chosen based on the results of these distribution tests. We used existing scales and recommended cut-offs which have been previously validated (Hotopf et al., 2006; Fear et al., 2010).

2. **Avoid the word “dearth,” since it is so overused**

Thank you for pointing this out. This statement has now been amended to avoid use of the word "dearth" (see line 92-93). This section now reads as:

“There is limited research on the prevalence of accidents, which did not result in death, in the UK military.”

We thank the editors and reviewers for their comments and advice regarding this paper. We hope that you find that these revisions address the reviewers’ comments suitably.
